# Lean Implementation for Graduating Optimally Controlled Stable Type 2 Diabetics from Endocrine Specialty Clinic Back to Primary Care

**DOI:** 10.1007/s11606-022-07767-z

**Published:** 2022-08-29

**Authors:** Lynn A. Burmeister, Peter G. Harper, Alvina Brueggemann, Patricia Adam, Mary Logeais, Kelly Schechter, Katie Duncan, Holly Boyer

**Affiliations:** 1 Division of Diabetes, Endocrinology & Metabolism, Department of Medicine, University of Minnesota, Minneapolis, MN USA; 2 Department of Family Medicine and Community Health, University of Minnesota, Minneapolis, USA; 3 M Health Fairview/University of Minnesota Physicians, Minneapolis, USA; 4 Division of General Internal Medicine, Department of Medicine, University of Minnesota, Minneapolis, USA

## Abstract

**Introduction:**

Endocrine specialty clinics (SCs) are occupied by a high percentage of stable follow-up patients, limiting access to new patients with greater needs.

**Aim:**

Feasibility project to improve access to diabetes SC by reducing the number of stable optimally controlled follow-up type 2 diabetic patients.

**Setting:**

M Health Fairview (MHFV), a hybrid network of University of Minnesota academic and Fairview Health community hospitals and clinics with affiliated providers.

**Program Description:**

A team-based lean methodology quality improvement graduation program including medical assistants, nurses, physicians, and a compact with primary care (PC) was used to identify within the Endocrine clinic population the graduation-eligible optimally controlled stable type 2 diabetic patients, acclimate them to the graduation concept, engage in shared decision-making, and transition them back to PC with a warm hand-off and graduation certificate.

**Program Evaluation:**

Seventeen percent (58/341) of eligible patients with optimally controlled diabetes graduated by 6 months, ranging between 0 and 83% per week.

**Discussion:**

The innovation and feasibility of opening SC access through the use of a team-based graduation program to transfer stable diabetes patients back to their home clinic was demonstrated. This innovation has the potential to support health system triage of new patients to limited access specialty care.

**Supplementary Information:**

The online version contains supplementary material available at 10.1007/s11606-022-07767-z.

## INTRODUCTION

As academic specialty clinics (SCs) are becoming part of large health systems across the USA, their population health missions are expanding as they are being asked to serve the needs of the system. Efforts to improve system-wide diabetes population health require both system- and patient-level approaches.^1^ This might include changes in the health system’s triage and use of limited resources.

Lean management strategy involves elimination of waste and improving processes for added value.^2^ The rising numbers of referrals to SCs have added to the perception of a specialist shortage and limited access.^3,4^ Yet, office-based specialist activity often includes services that could be managed in PC settings,^4^ wasting specialty time-space access.

Our system experienced a SC referral backlog with first appointments months out. Root cause analysis identified poor access due to the large number of chronic patients occupying the SC schedule (Supplement Figure 1). Only 8% of endocrine visits were for new patients. Most templated schedules were filled by every 3 to 6-month follow-up of the same patient, yet many of these follow-up patients already had optimally controlled diabetes with few changes made in their care plan. The system waste of limited specialty resources and timeliness of access to diabetes SC for higher acuity patients might be improved if optimally controlled stable patients were not chronically followed there.

We report an innovative graduation program utilizing a team-based approach and a collaborative agreement (compact) to “graduate” stable patients with type 2 diabetes back to PC. We demonstrate the feasibility of this approach as a first step to addressing waste of limited specialty resources and suboptimal specialty access.

## SETTING AND PARTICIPANTS

This program was implemented in the endocrine SC within the MHFV multi-specialty ambulatory Clinics and Surgery Center. MHFV is an academic health system partnership between the University of Minnesota and Fairview Health. The system comprises a hybrid network of academic and community hospitals and clinics with affiliated providers, using the Epic™ electronic medical record (EMR). The SCs serve this referral network.

The Transforming Clinical Practice Initiative^5^ implemented by Vizient sponsored Impact, a 5-month program designed to increase performance improvement capability at the practice level by using Lean methodology focusing on diabetes through the lens of team-based care.^2^ The graduation program team was one of four Impact Program teams formed to develop, implement, and monitor the interventions.^6^ The team included endocrinologists, primary care physicians (PCPs), nurses from specialty and PC, medical assistants (MAs), administrators, Epic™ builders, informaticists, and quality improvement (QI) specialists.

## PROGRAM DESCRIPTION

Our first Plan-Do-Study-Act (PDSA) cycle demonstrated the difficulty of informal efforts at returning stable diabetics to PC. Suggesting this to patients resulted in little to no change. Likewise, endocrinologists were uncomfortable broaching the topic of discharging even the stable patients from the SC, given lack of tools or system support. Finally, many patients were unable to identify, didn’t have, or had stopped seeing their PCP. Therefore, the graduation program team was assembled and reviewed the current state, designed and vetted the future process, guided PDSA cycles, and created educational materials tailored to each role. The team worked regularly on program development PDSAs for 6 months prior to the start of the intervention.

### Intervention Tools Development (Table 1)

EMR tools were developed to identify graduation-eligible patients. A “Graduation Button” was added to the check-out section of the EMR. A compact with the PC network was co-developed to define expectations, care transition needs and the specific necessary elements of the transition letter for returning patients to comprehensive PC.

**Table 1:** Key Innovation Elements

Innovation element	Impact of innovation element
Publicly announced goal-directed specialty optimization program with expectation to graduate back to primary care (on lobby TV screens)	Culture change for both patients and providers
EMR schedule check-mark indicator if graduation eligible criteria are met	Ease of identification of graduate eligible patients can be seen by whole team who can support the process repeatedly over time
Using the term “graduation” instead of discharge	Culture change for both patients and providers; positive emphasis on accomplishment
Graduation certificate	
Graduation Button	Removes patient from the diabetes clinic roster. Avoidance of future confusion in rescheduling back to diabetes clinic or prescription refill responsibilities
Compact with primary care	Collaborative system level change designed to meet the needs of both specialty and primary care settings
Structured graduation transition letter	

### Intervention Development Process

Patients with optimally controlled type 2 diabetes were graduation eligible. Optimally controlled type 2 diabetes was defined by the Minnesota Community Measure (MNCM) Optimal Diabetes Care criteria which include all of the following: HgbA1c < 8.0, blood pressure < 140/90, a non-tobacco user, and, if indicated, statin and aspirin use.^7^ Type 1 diabetic patients were excluded.

The graduation targets were based on historical endocrinology clinic statistics. In the previous year, the endocrinology clinic saw 2134 type 2 diabetic patients. Eight hundred twenty-eight (39%) met the graduation-eligible criteria. The target was to graduate 5% or 41 (41/828) patients back to PC during the 6-month intervention from 6/19/2019 until 12/31/2019.

### Changing Culture

In the clinic waiting room, digital education socialized patients to the concept of achieving goals leading to graduation (Supplement Figure 2). New patients were asked to identify their PCP as a requirement for scheduling. Follow-up patients were reminded of the need to establish or maintain PC. Specialty providers were encouraged to set goal-directed expectations for all patients for continuation in SC, anticipating return to PC once the goals were met.

### Intervention

Graduation was accomplished using a team-based care approach and a clearly defined process.

Before daily clinic sessions, graduation-eligible patients, denoted with a checkmark on the provider schedule, were listed on an easily visible white board to alert staff for pre-visit planning huddles. MAs introduced graduation during the rooming process or in other pre-visit communications. Endocrinologists would then engage in shared decision-making with patients. If all agreed, they were “graduated” and the transition would begin. The MA would present a Graduation certificate (Supplement Figure 3) and provide an After Visit Summary with graduation information (Supplement Figure 4), and the RN would provide a warm hand-off call to the PC clinic RN to coordinate care and to schedule the next appointment. A letter to the PCP (Supplement Figure 5) indicated final instructions and the patient was provided with a year of diabetes medication refills. Providers were given necessary program education at monthly faculty meetings and clinic staff were educated at daily and weekly staff huddles.

A thermometer diagram graphic of graduated patients was posted in the SC for visual reminder and discussion. Weekly clinic staff huddles including clinic leadership, MAs and RNs were used to review data and to debrief.

The main outcome measure was the percent of patients “graduated” back to PC. The percent of graduated patients with a warm hand-off and appointment scheduled at PC was also monitored. Reasons for non-graduation were indicated on the in clinic white board and tracked on a Pareto document. Balancing measures included patient and provider satisfaction, and the number of patients who were referred back to the Endocrine SC after graduation.

Analysis consisted of simple counts and percentages of graduated patients and hand-off elements. The study was determined exempt by the Institutional Review Board at the University of Minnesota.

## PROGRAM EVALUATION

Three hundred forty-one patient visits were graduation-eligible between 6/19/19 and 12/31/19. On average, 12 patients per week met eligibility criteria (range 3–24, Supplement Figure 6). Between 0 and 83% of eligible patients graduated each week, with a mean of 17% per week (SE 3.2). Run chart analysis showed significant variability of the percentage of eligible patients graduated over the 6 months of analysis, without apparent improvement trend over time (Supplement Figure 6). Within 4 months, 12% (41/341) were graduated, and this increased to 17% (58/341) by 6 months. Of the graduated patients, 93% (54/58) had a warm hand-off and a written notice to PC, and 57% (33/58) had an appointment scheduled with PC within 3 months of graduation.

Many patients were not graduated. Reasons for non-graduation included change in diabetes medication or dose (26%, 72); comorbid endocrine conditions (19%, 53); increase in HbA1C (16%, 46); patient refusal/not ready yet (10%, 29); 1–2-year follow-up with another MD (5%, 13); recent hospitalization (1%, 2); no show (2%, 7); other (4%, 14) (including no PCP, pregnancy, insulin pump, complex patient, transplant); and unknown (16%, 46) (Supplement Figure 7).

## DISCUSSION

A team-based Graduation program including a compact with PC to graduate stable diabetic patients back to PC was successfully designed, implemented, and sustained in the Endocrine SC. Within 4 months of implementation, the goal of graduating 5% of the stable diabetes patients back to PC was accomplished. This increased to 17% (58) by 6 months, demonstrating feasibility to open access to the Endocrine clinic for the care of new uncontrolled diabetes patients.

### Why This Is Important

Timely access to SC for PC patients can be critically important both for patient health and for PCPs with endocrinology-related dilemmas. With only 8046 US endocrinologists^8^ and 10% of the US population with a diagnosis of diabetes, mismatch between the inadequate endocrine workforce^9–11^, and a high clinical demand might result in backlog and long wait times for diabetes SC care. More efficient (less wasteful) matching of provider skill level to diabetes care needs might improve the timeliness of access to specialty level diabetes care. This could be accomplished in two ways: (1) Optimizing team-based care within diabetes clinics with more Advanced Practice Providers (APPs) and Certified Diabetes Educators (CDEs) or (2) Optimizing cooperation with PC. Engaging with PC has a potentially bigger impact given its greater population level reach. This was the approach taken in this paper.

### Changing Culture and Expectations

Achieving optimal health system efficiency relative to specialty use and access involves setting and changing culture at multiple levels, including expectations of patients, referring physicians, diabetes specialists. During the project, we encountered several cultural issues that needed to be addressed in our setting and will likely be encountered by others.

#### Team-Based Care

Teamwork is foundational to optimal diabetes care^2^ including the use of endocrinologists, SC APPs, CDEs, and PCPs, each bringing unique skills and potential for diabetes care improvement. Unfortunately, endocrinologists found that patients did not always agree to see team members other than their endocrinologist.

#### Primary Care in an Academic Multispecialty System

Recognition of PCPs as key members of the diabetes team, or even as experts in comprehensive care, had not been systemically acculturated either at the patient or the provider level. Formally bringing PCPs into the team with specified roles and processes (compact) is an important shift within an academic health system culture.

#### Patient Expectations

Patient resistance was the explicit reason for non-graduation in a minority of patients (10%). Endocrinologists sometimes reported that patients expressed fears of “break up” or abandonment by the diabetes care physician.

### Expected Adverse Consequences

The Graduation team discussed and accepted that improving SC access for poorly controlled diabetics may reduce MNCM SC scores, making the receiving SC appear less effective than competing SCs using the former model retaining their well-controlled diabetics. Narrow focus on these scores at the clinic level, rather than the system level, may present a barrier to successful graduation.

### Lean Process

A clinic staff not trained in QI was able to harness Lean principles and tools in order to achieve change in a short time interval. This included deliberate and formal interactions; consistent and visible leadership; frontline team engagement; team members from both endocrinology and PC; the use of QI specialists; and regular meetings with the entire team. Education on the scarcity of endocrine access, targeting referrals for more complex/difficult to manage patients, and encouraging other avenues of collaborative care, such as E-consults was foundational.

### Limitations

Due to the pandemic, we were not able to demonstrate the improvement in access to the Endocrine SC for new patients (Supplement Figure 8), nor obtain the balancing measures of patient and provider satisfaction or the number of patients who were returned to SC after graduation. This is a significant limitation. It is expected that access will be improved through graduating stable patients, but this will lag far behind the graduation curve. Specifically, 2 or 3 stable follow-up patients (which have shorter appointment length) need to graduate to open one potential new patient space (which has longer first appointment length). Likewise, patient and provider satisfaction, return to endocrine SC after graduation, and diabetes population control outcomes will also significantly lag behind graduation dates. This would be important to address in future work. Likewise, addressing the reasons for not graduating patients would be important in the future.

### Summary Statement/Future Work

In summary, using team-based Lean methodology, including a compact with PC, our innovative program graduated stable diabetes patients back to their PC clinic, demonstrating feasibility to open new specialty patient access. This feasibility would apply to all types of SCs. Future work needs to address the identified cultural barriers to further improve the graduation rate, measuring the impact of graduation on clinic access, and testing whether less stringent criteria could be successful in graduating patients back to PC earlier.

## Supplementary information

ESM 1 (DOCX 223 kb)
