# Supplementary material for: Lean Implementation for Graduating Optimally Controlled Stable Type 2 Diabetics from Endocrine Specialty Clinic Back to Primary Care
Source: J Gen Intern Med. 2022 Aug 29;37(15):4004–7. doi: 10.1007/s11606-022-07767-z (PMC9640490; doi:10.1007/s11606-022-07767-z)
Supplement: Supplementary file 1 — (DOCX 223 kb) [file 11606_2022_7767_MOESM1_ESM.docx]

**Supplemental figure 1.** The 5 Whys

New patients can't get into the endocrine clinic in a timely manner.

1. The wait for a new patient is often over 28 days. Why?

2. We don't have enough NEW patient slots. Why?

3. Most appointment slots are filled in advance by existing return patients. Why?

4. We keep our existing patients and do not refer them back to primary care. Why?

5. We have never had a process for providers to recognize and graduate patients. Also, our patients love us and value the relationship they have with us - and we haven't felt comfortable or

supported because we do not have a systematic approach in severing that tie with the patient and sending them to primary care.

**Supplement figure 2. Patient-facing lobby television screen education material**


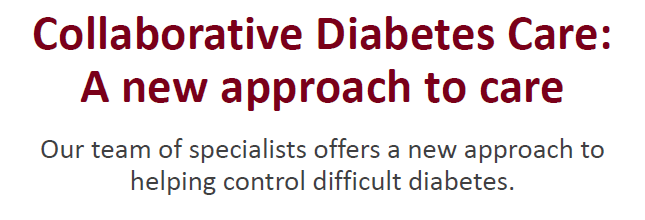


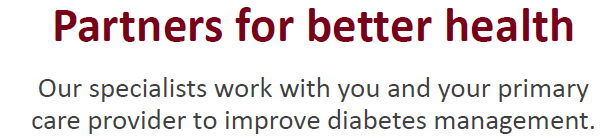


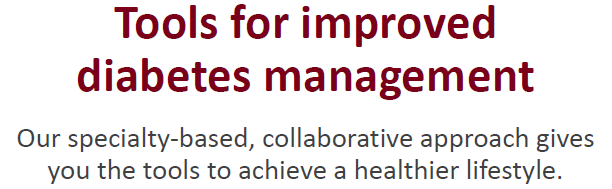


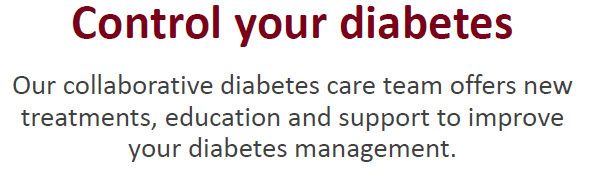


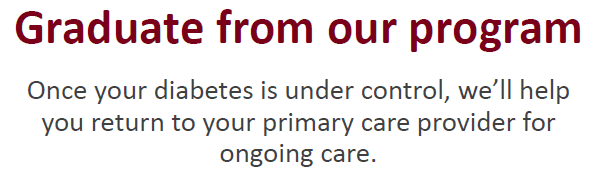


**Supplement figure 3.** Graduation certificate


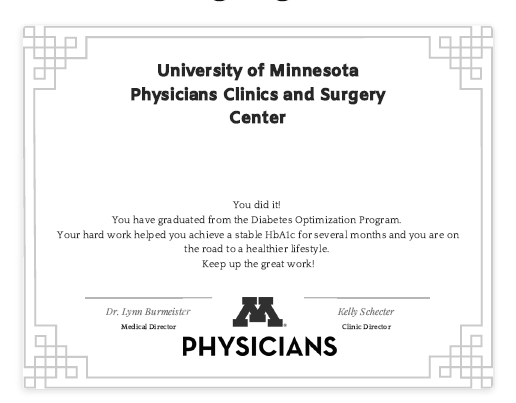


**Supplement figure 4.**  After visit summary dot phrase

You have earned this graduation by achieving diabetes related goals and stability.

It has been a pleasure serving you.

Please use the lessons learned in the diabetes clinic as a base on which to build a lifetime of better health and wellness.

You should continue to regularly follow up with your primary care provider for diabetes management.

Please schedule an appointment with your primary care provider within the next 3 months.

We have refilled all of your diabetes-related medication for the next year.

Future changes and refills should come from your primary care provider.

The diabetes care team remains available to you for future consultation should you and your doctor have new questions or concerns.

**Supplement figure 5.** Template for letter to primary care doctor

Dear Doctor:

Thank you for referring your patient “patient name” to the MHealth Endocrinology clinic for evaluation/ treatment of diabetes mellitus type 2. At this point, “patient name” has reached a stable level for blood sugar-related diabetes care and would be best served in your primary care office. “Patient name” is aware of our recommendation and was instructed to schedule a visit with you within the next 3 months and to contact you with any future concerns.

Our recommendations for ongoing care are:

{Diabetes Medication Types:141714}

{Blood Pressure Medications Bloom:124249}

ASA 81 mg/day if indicated

{STATINBAE:105764}

I have updated the Diabetes related Problem List (in the Overview), noting we are returning the care of “patient name” to you. We would appreciate it if you helped us keep the Overview section up to date, so it is clear who is managing “patient name's” Diabetes.

I have also provided refills for the next year to help with the transition back to your team. After this, you should continue to provide the necessary refills.

Routine diabetes related follow up for “patient name” may continue under your direction, and should include the following:

Yearly labs: lipids, creatinine, potassium, urine albumin/creatinine ratio

DM visit with HbA1c every 3-6 months. The target HbA1c for this patient is ***

Diabetic foot exam yearly minimum, ideally every visit

Ophthalmology exam yearly or more frequent if deemed necessary by the patient's ophthalmologist

Routine follow up in diabetes education with certified diabetes educators (CDE) including either/or both RD CDE and RN CDE, as indicated. Typical eligibility includes:

3 hours/year of medical nutrition therapy

2 hours/year of nursing education for previous diabetes diagnosis

Feel free to contact us with any future questions or concerns that might arise with “patient name” diabetes care. Alternatively, we would be happy to see “patient name” again, as needed, if questions or concerns arise, including rising hemoglobin A1c, concern for new diabetes or medication related complications or need for adjustment.

Sincerely,

Dr. *** from the Diabetes team

References:

Standards of Medical Care in Diabetes-2019 Abridged for Primary Care Providers

Supplement figure 6. Run charts of the number of graduation-eligible patients and the percent of eligible patients actually graduated per week during the intervention period from 6/19/19-12/13/19


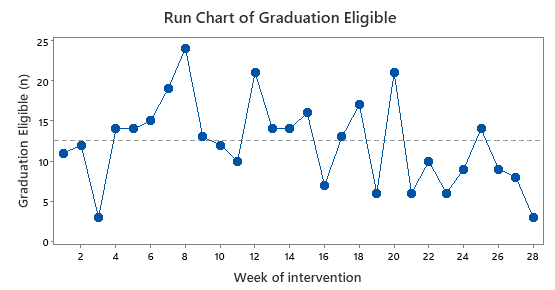


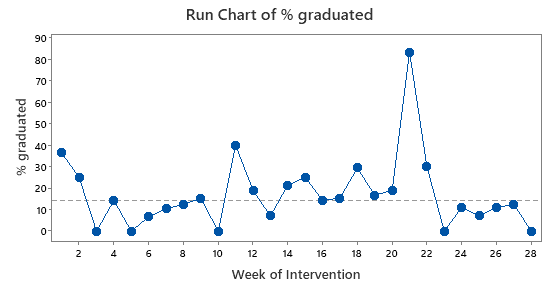


Supplement figure 7. Pareto graph of the reasons for non-graduation


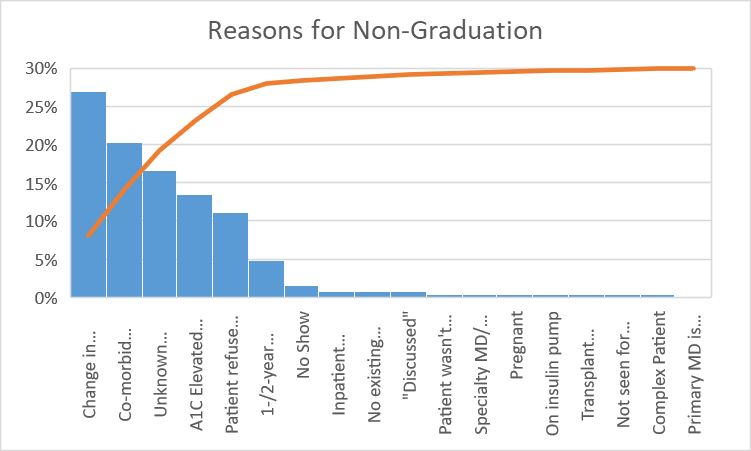


Supplement figure 8. Run chart of New Diabetes access during the preintervention, intervention, post intervention, and COVID pandemic periods


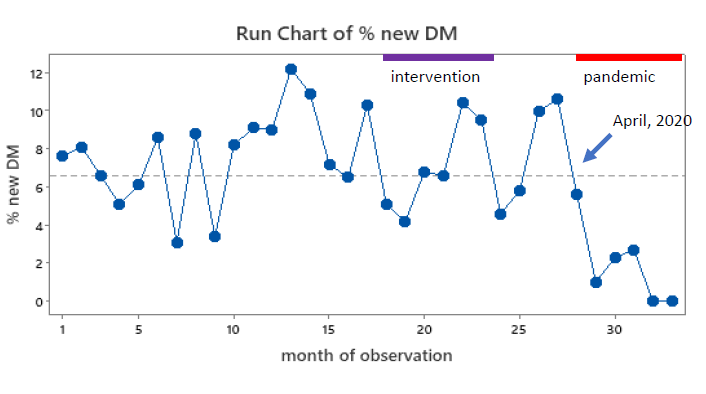
Percentage of diabetes clinic encounters accessing new patients. The pre-intervention months (1-18) range from January 2018 to 6/30/2019. The Graduation program intervention ran from 6/19/2019-12/31/2019 (months 19-24). Due to the COVID 19 pandemic, the clinic abruptly transitioned from face to face to virtual visits starting mid-March 2020. Visit types were created for virtual visits which did not distinguish between new diabetes or other diagnosis, partially explaining the significant drop in % of new diabetes encounters in the early months of the pandemic. Percentage of new diabetes visit types per week: pre-intervention 7.7 +/- 0.6; intervention/post 7.4 +/- 0.8, pandemic 1.9 +/- 0.9.
